# *In vitro* Assessment of Chemical and Pre-biotic Properties of Carboxymethylated Polysaccharides From *Passiflora edulis* Peel, Xylan, and Citrus Pectin

**DOI:** 10.3389/fnut.2021.778563

**Published:** 2021-12-03

**Authors:** Yongjin Sun, Yuan Guan, Hock Eng Khoo, Xia Li

**Affiliations:** ^1^Department of Bioengineering, College of Chemistry and Bioengineering, Bioengineering Program, Guilin University of Technology, Guilin, China; ^2^Guangxi Key Laboratory of Electrochemical and Magnetochemical Functional Materials, College of Chemistry and Bioengineering, Bioengineering Program, Guilin University of Technology, Guilin, China

**Keywords:** chemical modification, growth curve, passion fruit, probiotic, functional group

## Abstract

This study aimed to determine the carboxymethylation effect of crude water-soluble polysaccharides of *Passiflora edulis* peel (WPEP), xylan (XY), and citrus pectin (CP). Their chemical and pre-biotic properties were also determined. The polysaccharides were carboxymethylated by reacting with chloroacetic acid and sodium hydroxide. The carboxymethylated and non-carboxymethylated polysaccharides were also used as pre-biotics to study the growth pattern of selected intestinal microflora. These polysaccharides substituted the glucose solution in culture media for culturing *Lactobacillus brevis* GIM1.773, *Lactobacillus plantarum* GIM1.19, *Lactobacillus delbrueckii* subsp. *bulgaricus* GIM1.155, and *Streptococcus thermophilus* GIM1.540. The results showed that the carboxymethylated polysaccharides c-XY, c-CP, and c-WPEP, had substitution degrees of 0.682, 0.437, and 0.439, respectively. The polysaccharides demonstrated resistance to digestion in the simulated human digestive models. The resistance to digestion was enhanced by carboxymethylation, especially the carboxymethylated CP and WPEP. The results also showed that the pre-biotic activities of the polysaccharides increased after carboxymethylation. The c-XY had a better pre-biotic effect than XY and the other carbohydrate samples. The findings suggested that carboxymethylated polysaccharides may be developed into novel pre-biotics and nutraceuticals that could promote growth of the probiotic strains.

## Introduction

Pre-biotics are defined as substrates that could selectively promote growth and activity of the host microorganisms. They are non-digestible oligosaccharides that have a beneficial effect on the human gut. The substrates also maintain the balance of intestinal microecology (1, 2). These oligosaccharides are food for gut microflora, such as lactobacilli or bifidobacteria. These bacteria inhabit the human intestinal tract, and they are responsible for regulating fat storage and biosynthesis of essential vitamins (3). Pre-biotics are metabolized into lactic acid and short-chain fatty acids in the large intestine. These substances promote the growth of intestinal bacteria and improve the physiological health of the host. Regular consumption of pre-biotics is essential for maintaining good health and regulating intestinal microflora (4). Studies have shown that the experimental mice fed pre-biotics had improved intestinal microflora. Pre-biotics not only help to treat obesity but also improve the host's immune system and prevent the development of diseases like type 2 diabetes mellitus, irritable bowel syndrome, and colorectal cancer. These oligosaccharides can also indirectly regulate cardiovascular diseases (5).

Polysaccharides are potent sources of pre-biotics. They have antiviral, immuno-enhancing, hypoglycemic, antioxidation, and antitumor effects in addition to the pre-biotic properties (6–8). The substances also effectively promote the growth of intestinal microflora and increase short-chain fatty acid levels (9, 10). Literature showed that the polysaccharides extracted from the citrus peel (11), bamboo shoots (12), *Ganoderma lucidum*, and *Poria cocos* (13) exhibited pre-biotic potential. However, the pre-biotic activities of carboxymethylated polysaccharides were yet unknown. The consumption of sulfated polysaccharides from marine seaweeds as pre-biotics showed anti-inflammatory effects and prevented peptic-ulcer disease and gastrointestinal disorders (14). The disease-prevention mechanism is related to the blocking of the leucocyte adhered to the epithelium of blood vessels. The polysaccharides also prevented the migration of these cells to the inflammation sites. However, the biological activities of these polysaccharides are limited by their low solubility. Many scholars have also attempted to chemically modify the structures of polysaccharides to improve their physicochemical properties and bioactivities (2, 15).

The chemical modification of polysaccharides, especially carboxymethylation, has recently drawn wide attention. The carboxymethylation technique has been used to improve the physicochemical properties and bioactivity of plant polysaccharides. The carboxymethylated polysaccharides exhibited immunoregulatory, oxidation, and antitumor effects (16–19). A study on characterization of carboxymethylated xylan has been done previously, and the structural information has been obtained using ^13^C nuclear magnetic resonance (20). The structural characteristics of carboxymethylated pectin had also been studied using Fourier-transform infrared spectroscopy (FTIR), X-ray diffraction, and thermogravimetric analysis (21). Moreover, physicochemical characteristics of polysaccharides extracted from passion fruit peel had been performed (22). As no previous study has been done on carboxymethylation of polysaccharides extracted from passion fruit peel, this study is, therefore, aimed to fill such a gap.

Currently, the valorization of natural resources and utilization of renewable energy resources leads to universal sustainability. The by-products of food processing are the new sources of sustainable food that are renewable and eco-friendly (23). Fruit peels or pericarps are some of the polysaccharide-rich wastes. Polysaccharides have been extracted from citrus and passion fruit peels. The use of polysaccharides from fruit peel provides a new idea for waste utilization. These polysaccharides are potent functional foods. Functional food is one of the most promising and fastest developing health foods in the food industry. Pre-biotics have been widely studied and commercially explored. The data on pre-biotic effects of carboxymethylated polysaccharides are also limited. Therefore, these polysaccharides have great application potential in the functional food and medical industries.

## Materials and Methods

### Chemicals and Reagents

Fructooligosaccharide (FOS) was purchased from Guangdong Guanghua Chemical Factory Co., Ltd (Guangdong, China); trypsin, pancreatin solution, bile salt, and α-amylase were purchased from Qingdao Hi-tech Park Haibo Biological Technology Co., Ltd. (China). All other chemicals and reagents were obtained from Xilong Chemical Co., Ltd. (Guangdong, China). All chemicals and reagents used in this study were of analytical grade.

### Sample Preparation

The analytical grade of xylan (XY) was obtained from the Guangxi Institute of Botany (Guilin, China). The XY sample was prepared according to the method described by Miao et al. (24). Citrus pectin (PC) was purchased from the Shanghai Yuanye Biotechnology Co., Ltd. (Shanghai, China), and the WPEP sample was prepared according to the method described by Guan et al. (22). *Lactobacillus brevis* GIM1.773*, L. plantarum* GIM1.19, *L. delbrueckii* subsp. *bulgaricus* GIM1.155, and *Streptococcus thermophilus* GIM1.540 were obtained from the Guangdong Institute of Microbiology (Guangzhou, China).

### Preparation of Carboxymethylated Polysaccharides

The chloroacetic acid–sodium hydroxide reaction procedure was adapted from the method described by Wang et al. (25). An exact 240 mg of XY, CP, and WPEP was separately dissolved in 20 mL of 20% sodium hydroxide (NaOH) solution and 50 mL isopropanol and stirred for 3 h in an ice-water bath to obtain a uniform suspension. Next, 6.0 g chloroacetic acid was mixed with 50 mL of isopropanol until complete dissolution was achieved. Then, 20 mL of 20% NaOH solution was added to the mixture and heated for 3 h in a water bath of 60°C. The conical flask was cooled to room temperature, and the solution pH was adjusted to 7 by adding 1 M hydrochloric acid (HCl). Finally, the mixture was dialyzed for 24 h with tap water, concentrated, and freeze-dried to obtain carboxymethylated polysaccharides. The carboxymethylated XY, CP, and WPEP were named c-XY, c-CP, and c-WPEP, respectively.

### Determination of Chemical Composition

The phenol-sulfuric acid method was used to determine the total sugar content of the polysaccharide samples (26). Briefly, 1 mL of 0.1 mg/mL sample was added with 0.5 mL of 6% phenol reagent and 2.5 mL of concentrated sulfuric acid. The reacting solution was placed into a HH-W420 water bath (Shanghai Fangrui Instrument Co., Ltd., Shanghai, China) at 100°C for 10 min and then cooled to room temperature. The changes in absorbances at 490 nm were determined, and the total sugar content was calculated based on the glucose standard curve (0–1.0 mg/mL).

The m-hydroxydiphenyl method was used to determine the galacturonic acid content of the polysaccharide samples (27). In brief, 400 μL sample solution (0.1 mg/mL) was added with sulfamic acid (0.39 mg/mL), homogenized, and then added with 2.5 mL of concentrated sulfuric acid. The mixture was placed in boiling water for 20 min. After cooling to room temperature, 40 μL of m-hydroxydiphenyl reagent was added to the solution mixture and kept at room temperature for 15 min. The absorbance was measured at 595 nm. The standard curve was plotted based on different concentrations of galacturonic acid (0–400 mg/mL).

Bradford method was used to determine the total protein content of the polysaccharide samples (28). Briefly, 1.0 mL of the sample solution (0.1 mg/mL) was added with 4 mL of Coomasie Blue reagent and then placed at room temperature for 5 min. Bovine serum albumin (0–1.0 mg/mL) was used as the protein standard. The absorbance was measured at 595 nm.

### Fourier-Transform Infrared Spectroscopy

Exactly 1.0 mg of the freeze-dried sample was mixed with 100 mg potassium bromide, pulverized, and then pressed into disks. The Nicolet iS10 FTIR spectrometer (Thermo Scientific, Waltham, USA) was used to obtain the absorption spectra of compounds. The wavelengths used ranged from 4,000 to 400 cm^−1^.

### Scanning Electron Microscopy

The polysaccharide samples (1 mg/mL) were dissolved in deionized water and freeze-dried to produce sample specimens. A SU5000 field emission scanning electron microscope (SEM) (Hitachi, Tokyo, Japan) was performed to observe the morphology of the polysaccharide samples at 20°C with an acceleration voltage of 5 kV and magnification of 300×.

### Determination of Degree of Substitution

The degree of substitution (DS) was determined by the neutralization titration (29). Briefly, 10 mg polysaccharide was diluted with 3 mL 70% methanol, then 10 mL distilled water and 5 mL 0.5 mol/L sodium hydroxide were added, and finally titrated with 0.1 M HCl until the color of phenolphthalein in the mixture faded. The carboxymethylation degree (A) of the polysaccharide samples was determined as follows:


(1) $$\begin{array}{l} \text{A}=\frac{V_{0}M_{0}\;-\;\left(V_{2}\;-\;V_{1}\right)M}{W} \end{array}$$

where V_0_ is the amount of NaOH (mL) added, V_1_ is the volume of HCl used to titrate the sample (mL), V_2_ is the amount of HCl (mL) used, M_0_ is the increase in the concentration of sodium hydroxide (0.5 mol/L), M is the concentration of HCl used to titrate the sample (0.1 mol/L), and W is the mass of the sample (g). The degree of substitution (DS) was calculated as follows:


(2) $$\begin{array}{l} DS=\frac{0.162A}{1-0.058A} \end{array}$$

### Hydrolysis Degree of Polysaccharides

#### Simulated Saliva Digestion

The digestion reagent of the simulated saliva was prepared by adding 0.764 g sodium chloride, 1.491 g potassium chloride, and 0.133 g of calcium chloride into distilled water. The total volume was increased to 1,000 mL, and the solution was adjusted to pH 6.9 with 1 M sodium bicarbonate. An exact 0.345 g α-amylase was then dissolved with 400 mL of the digestion reagent, magnetically stirred for 20 min, and finally filtered. The filtered was added with another 400 mL of the digestion reagent before adding 1 mg/mL sample solution at a ratio (1:1) and then placed in the water bath of 37°C to imitate the oral environment. The digesting samples were collected at 0 h and 0.5 h and then boiled for 5 min to inactivate the enzyme. The 3,5-dinitrosalicylic acid method and phenol-sulfuric acid were used to determine the reducing sugar and total sugar content. The degree of hydrolysis was calculated based on the formula as follows:


(3) $$\begin{array}{l} \text{Hydrolysis}\;\text{degree}\;(\%)\\ =\frac{\text{Hydrolyzed reducing sugar}}{\text{Total sugar - Non hydrolyzed reducing sugar}}\;\times \;100 \end{array}$$

#### Simulated Gastric Digestion

The experimental method was slightly modified from the method described previously (30–32). The buffer solution was prepared by adding 8.25 g disodium hydrogen phosphate monohydrate, 14.35 g monosodium phosphate, 8.0 g sodium chloride, 0.2 g potassium chloride, 0.1 g calcium chloride, and 0.18 g magnesium chloride hexahydrate with distilled water and diluted to 1,000 mL. The pH of the buffer solution was adjusted to 1, 2, or 3 with 1 M HCl solution. The sample weighing 100 mg was added to 10.0 mL of the buffer solution and placed in a water bath at 37°C for 6 h. An exact 1.0 mL of the sample solution was obtained at 4 and 6 h during the simulated gastric digestion to determine reducing sugar and total sugar content. The degree of hydrolysis was calculated using Equation (3).

#### Simulated Small Intestinal Digestion

The simulated small intestinal digestion was performed according to the method described previously (2, 31). The simulated small-intestinal juice was prepared by adding 5.40 g sodium chloride, 0.65 g potassium chloride, and 0.33 g calcium chloride into a conical flask, dissolved with distilled water, and diluted to 1,000 mL. Next, 13 mg trypsin, 100 mL of pancreatin solution (7%, w/w), 200 mL of bile salt (4%, w/w), and the juice solution were mixed before the pH was adjusted to 7 with 1 M sodium bicarbonate. Then, 1 mg/mL sample solution was mixed with the simulated small-intestinal juice at a ratio of 1:1. During the digestion, 1.0 mL of the digesting sample was separately collected at 4 and 6 h and then boiled for 5 min to inactivate the enzymes. The reducing sugar and total sugar content were determined. The degree of hydrolysis was calculated according to Equation (3).

### Preparation of Culture Media

The basal medium for culturing *S. thermophilus* was prepared by mixing peptone (5.0 g), yeast extract powder (10.0 g), calcium carbonate (1.0 g), dipotassium phosphate (2.0 g), glucose (15.0 g/L), cysteine (0.50 g), and Tween-80 (1.0 mL) in a beaker, and the mixture was heated until dissolution. The medium was topped up with distilled water to 1 L. The pH was adjusted to 6.5, and the solution was sterilized for 20 min at 121°C. The basal medium for the other strains was prepared by mixing tryptone (10 g/L), beef extract powder (10.0 g), yeast extract powder (5.0 g), ammonium citrate (2.0 g), dipotassium phosphate (2.0 g), manganese sulfate monohydrate (0.30 g), Tween-80 (1 ml/L), urea (15 g/L), and sodium acetate (5 g/L), and then heated until dissolution. The distilled water was then added to the mixture to obtain a total volume of 1 L. The pH was adjusted to 6.5, and the solution was sterilized. The experimental media were prepared by replacing glucose solution in the basal medium with XY, CP, WPEP, and the carboxymethylated polysaccharide samples.

### Pre-biotic Effect of the Carboxymethylated Polysaccharides

The pre-biotic effect of the polysaccharide samples was determined based on the microbial growth assay. The polysaccharide samples were added to the culture media as the only carbon source. The four probiotic strains used were *L. brevis, L. plantarum, L. delbrueckii subsp. bulgaricus*, and *S. thermophilus*. Different concentrations of the polysaccharide samples were first used to screen for the microbial growth-promoting effect. In brief, a 100 μL of probiotic solution (2 × 10^8^ CFU/mL) was pipetted to the culture medium and then cultured for 48 h at 37°C. The optical density (OD) values were determined by measuring the absorbance at a wavelength of 600 nm. The OD value denotes the optimal concentration of polysaccharides used for the growth of the probiotic strains. The microbials were then cultured at different incubation times (0, 4, 8, 12, 24, and 36 h) by applying the optimized polysaccharide concentration. The OD values of the cultures were measured, and the results were expressed as log CFU/mL. A linear regression equation was obtained for each probiotic strain. The optimal polysaccharide concentration of 3% (w/v) was chosen as the only carbon source, and FOS was used for comparison. The growth curves of the four probiotic strains cultured using the culture media containing different polysaccharide samples were plotted.

### Statistical Analysis

All data were expressed as mean ± standard error of the mean (n = 3). The statistically significant differences were determined between the different groups based on the analysis of variance coupled with Duncan's multiple range test and student's *t*-test. The statistical analysis was performed using SPSS 26.0 software. *P* < 0.05 was considered a statistically significant difference.

## Results and Discussion

### Chemical Composition Analysis

Three polysaccharides were used in carboxymethylation. They were XY, CP, and WPEP. The degrees of substitution for c-XY, c-CP, and c-WPEP were 0.68, 0.44, and 0.44, respectively (Table 1). XY had the highest degree of saturation in comparison with CP and WPEP. The results also showed that the carboxymethylated polysaccharide samples had a significantly lower total sugar content than the non-carboxymethylated samples (*P* < 0.05). Although the carboxymethylated polysaccharides had total protein content lesser than the non-carboxymethylated forms, no significant differences were found between the polysaccharide samples (*P* > 0.05). WPEP also had a significantly lower galacturonic acid content besides the total sugar and protein content. On the contrary, the c-XY and c-CP had a significantly higher galacturonic acid content than the non-carboxymethylated forms (*P* < 0.05).

**Table 1:** Chemical compositions of polysaccharide samples.

**Samples**	**Total sugar (%)**	**Total protein (%)**	**Galactoronic acid (%)**	**DS**
WPEP	54.23 ± 1.62^e^	2.98± 0.43^a^	39.43 ± 2.33^a^	
XY	94.43 ± 4.77^a^	1.49 ± 0.22^c^	4.32 ± 1.74^f^	
CP	87.25 ± 3.76^b^	1.33 ± 0.92^c^	6.72 ± 2.25^e^	
c-WPEP	39.46 ± 1.25^f^	2.48 ± 0.57^b^	33.23 ± 1.92^b^	0.44 ± 0.06^b^
c-XY	79.12 ± 3.62^d^	1.42 ± 0.14^c^	9.43 ± 1.43^c^	0.68 ± 0.02^a^
c-CP	79.63 ± 1.89^c^	1.12 ± 0.91^d^	7.66 ± 1.47^d^	0.44 ± 0.04^b^

*Data are presented as mean ± standard error of the mean of three replicates. Different lowercase superscript letters in the same column denote significant differences (P < 0.05)*.

**Figure 1:**
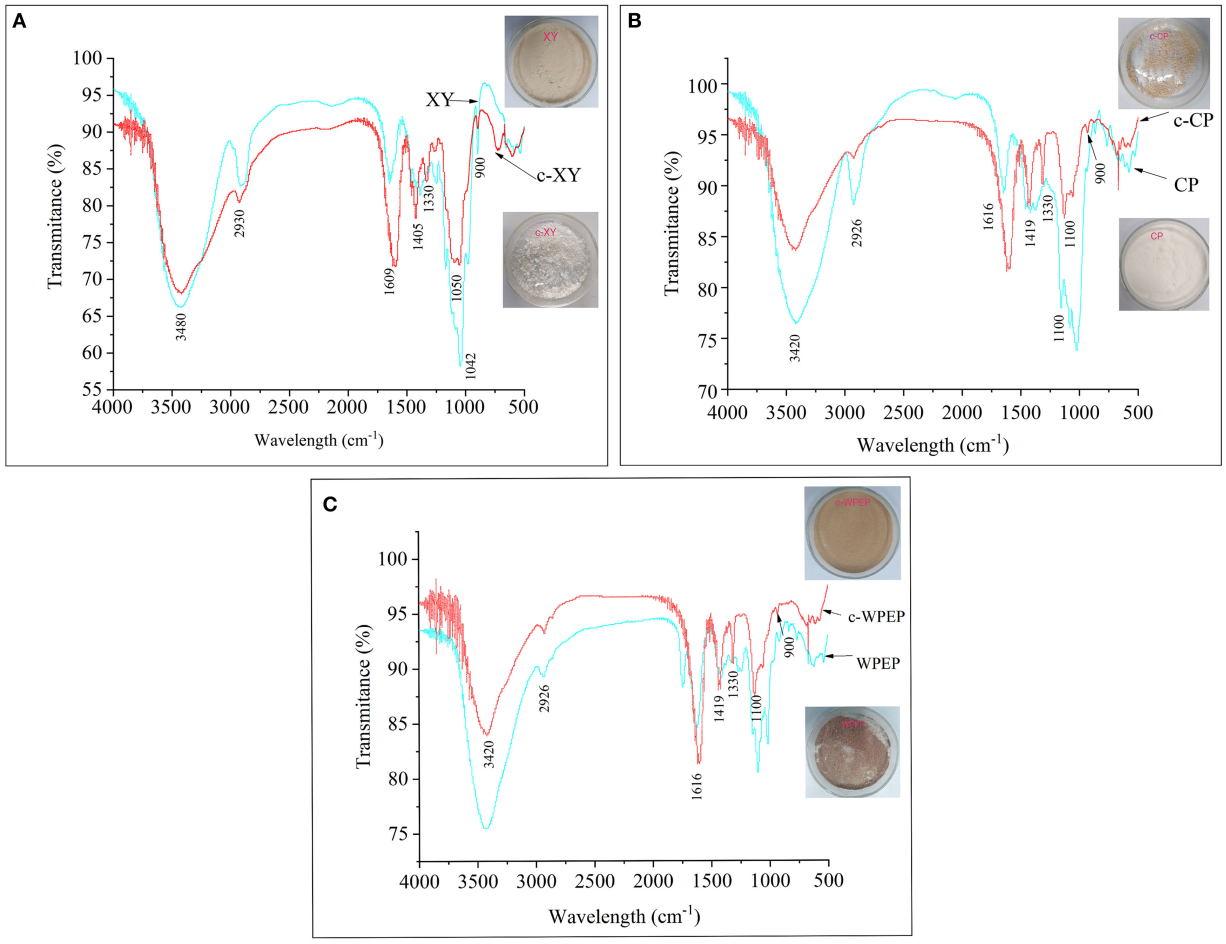
FT-IR spectra of **(A)** xylan (XY), **(B)** citrus pectin (CP), and **(C)** crude water-soluble polysaccharides of *Passiflora edulis* peel (WPEP) samples.

The XY was a fine light-yellow powder, CP was a white powder, and WPEP was a golden particle (Figure 1). The colors and appearances of XY, CP, and WPEP were remarkably changed after carboxymethylation. These changes indicated that the internal structure of the polysaccharides could have been modified chemically. Carboxymethylation of polysaccharides extracted from *Cyclocarya paliurus* showed a lower protein content than the non-carboxymethylated samples (33). Besides, the solubility of the polysaccharides increased after carboxymethylation (34). Moreover, the bioactivity of the c-XY improved (35).

The IR spectra of the c-XY and XY are shown in Figure 1A. The result showed that the broad peak at 3,480 cm^−1^ could be attributed to the stretching vibrations of the OH group. The IR peak at 2,930 cm^−1^ could also be attributed to the stretching vibration of the CH group. The result revealed C–O–C stretching vibrations at 1,330 cm^−1^. The peak spectra of c-XY close to 1,609 and 1,405 cm^−1^ also showed the characteristic absorption peaks of C=O and CO, respectively.

The IR spectra of the c-CP and CP are shown in Figure 1B. The broad peak at 3,420 cm^−1^ could be attributed to the stretching vibrations of the OH group. The IR peak at 2,926 cm^−1^ was due to the stretching vibration of the CH group in c-XY, and the peak at 1,330 cm^−1^ revealed C–O–C stretching vibrations of the carboxymethylated structure. The peak spectra of c-XY close to 1,616 and 1,419 cm^−1^ also showed the characteristic absorption peaks of C=O and CO, respectively.

The IR spectra of the c-WPEP and WPEP are shown in Figure 1C. The broad spectrum peak at 3,420 cm^−1^ could be attributed to the stretching vibrations of the OH group. Similar to c-XY and c-CP, the CH, C–O–C, C=O, and CO stretching vibrations were found for the c-WPEP. Also, the spectra of XY, CP, WPEP, c-XY, c-CP, and c-WPEP indicated typical characteristic absorption peaks of the polysaccharides at wavelengths of 1,100 and 3,500 cm^−1^.

The carboxymethylated polysaccharides had a lower peak height (broad peak) than the non-carboxymethylated forms. There was a sharp band at 900 cm^−1^, where it arose from the C1 group frequency or ring frequency. It was the characteristic of β-glucosidic linkages between the sugar units. The peak spectra of c-XY, c-CP, and c-WPEP close to 1,600 and 1,425 cm^−1^ also revealed the characteristic absorption peaks of C=O and CO, respectively. The findings indicated the successful carboxymethylation of the polysaccharide samples.

The carboxymethylation was performed using both aqueous and organic media. The use of organic medium has many advantages, including high reaction stability and degree of substitution. The substitution degree of XY was higher than CP and WPEP because the purity of XY was higher than these substances. CP and WPEP had the same substitution degree because the main polysaccharide in WPEP was pectin. Literature shows that the purple passion fruit peel has as high as 12.6% of pectin. The major monosaccharides of pectin were rhamnose, arabinose, and galactose (36). These monosaccharides had been confirmed as the major components of the water-soluble polysaccharides of passion fruit peel (37).

The biological activity of polysaccharides is greatly affected by the functional groups that exist in the molecular structures. The presence of functional groups in a polysaccharide determines the size and bioactivity of the polysaccharide. Also, the chemical modification of a polysaccharide introduces new functional groups to its molecular structure. The spatial structure influences bioactivity of the polysaccharide. The polysaccharide structure with flexural waves has higher bioactivity than the others, whereas the polysaccharide with wrinkle-shaped or stretchable ribbons has low bioactivity (38).

The sweetness of a polysaccharide is derived from the monosaccharide molecules. The high number of hydroxyl groups (–OH) of a polysaccharide denotes a high polysaccharide solubility in water and bioactivity (39). The sweetness of a polysaccharide is also attributed to the OH group. The carboxymethylation increased the intensity of stretching vibrations of the OH group. Therefore, the carboxymethylated polysaccharide could be sweeter than the non-carboxymethylated form.

### Scanning Electron Microscopy Analysis

**Figure 2:**
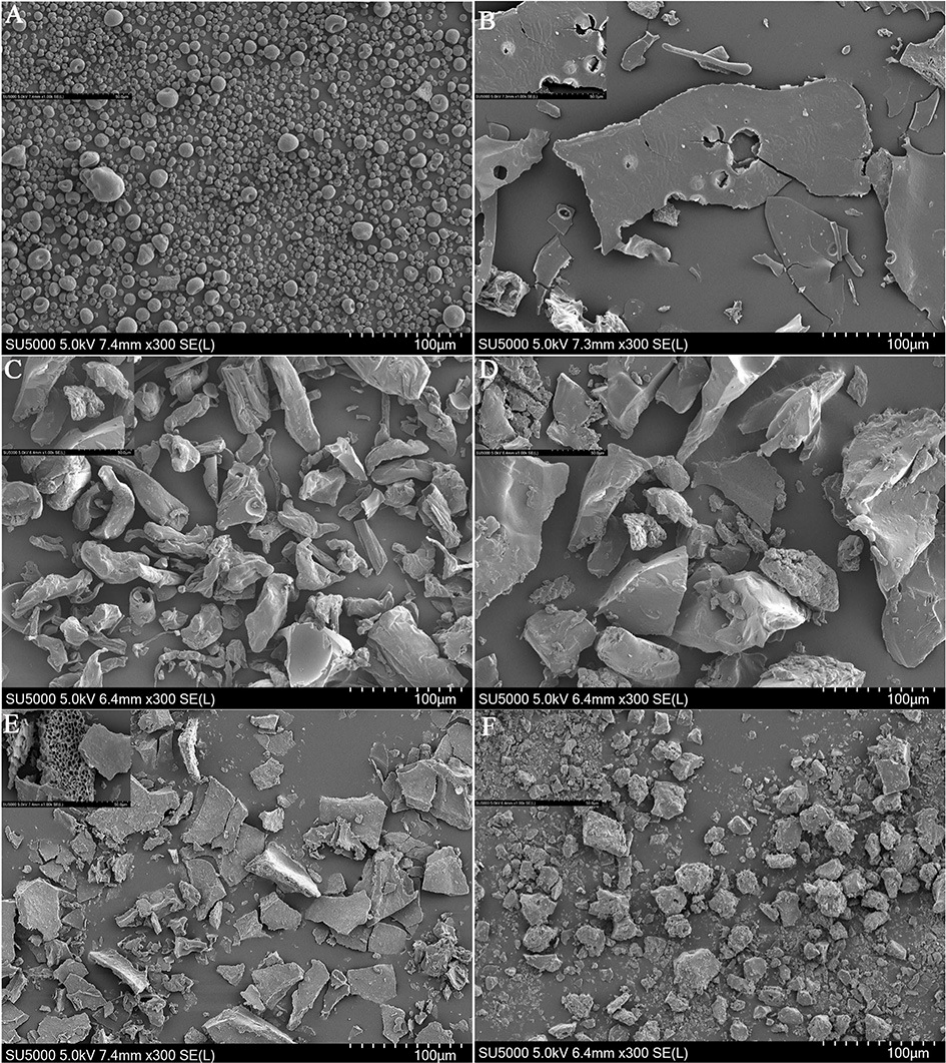
SEM micrographs of **(A)** xylan (XY), **(B)** carboxymethylated xylan (c-XY), **(C)** citrus pectin (CP), **(D)** carboxymethylated citrus pectin (c-CP), **(E)** crude water soluble polysaccharides of Passiflora edulis peel (WPEP) samples, **(F)** carboxymethylated crude water soluble polysaccharides oPfassiflora edulis peel (c-WPEP) samples; magnification factor: 300×.

The surface structures of the polysaccharides were observed by SEM (Figure 2). The result showed that the surface structure of WPEP was multiporous, mildly rough, and unbounded. The surface structure of CP was less flaky, mild fibrous look, and some were rod-like structure; the XY had a granular shape. The changes in the surface structures of the polysaccharide samples were observed after the carboxymethylation, especially the surface of XY became flaky. The WPEP became lumpy after the carboxymethylation. The size of the flaky structure of c-WPEP was also reduced. The rod-like shape was not seen in the c-CP. The findings confirm that the structural surface of these polysaccharides had been modified by carboxymethylation.

The surface structural differences between the polysaccharide samples were due to the variation in molecular structures and bondings (40). The WPEP could contain other glycans besides pectin. The CP used in this study had 65% purity. The pectin in the purified extract might be bound together with glycans like xylan, xyloglucan, and glucuronoxylan (41). However, carboxymethylation increased the roughness and irregularity of the surface structure of xylan, with hollows and embossment (42). Also, CP prepared from manosonication assisted extraction formed an amorphous, rough, and hard surface with surface cracking and particles stuck to the surface.

### Hydrolysis Degree of Polysaccharides

The study of resistance of the polysaccharide digestion was performed based on assays mimicking the human digestive tract. The digestion resistance was explored using simulated saliva, gastric juice, and small intestinal juice methods. The digestion resistance rate was determined based on the hydrolysis degree. The minimal differences among the polysaccharides and FOS in terms of the degree of hydrolysis are shown in Table 2. The results showed that most polysaccharide samples had a moderate degree of resistance to digestion. The hydrolysis degrees of c-WPEP assessed by the simulated saliva, gastric juice, and small intestinal juice methods were lower than the WPEP (*P* < 0.05). It showed that the carboxymethylation of WPEP increased digestion resistance in the human digestive tract. The hydrolysis degrees of c-XY and c-CP were not significantly improved (*P* > 0.05). The hydrolysis degrees of XY and CP after 4 h of hydrolysis using the simulated small intestinal juice test were significantly higher than the carboxymethylated samples (*P* < 0.05).

**Table 2:** Hydrolysis degrees (%) of polysaccharides in simulated salivary, gastric, and intestinal conditions.

**Samples**	**Simulated**	**Simulated gastric**						**Simulated intestinal**	
	**saliva**								
	**0.5 h**	**4 h**			**6 h**			**4 h**	**6 h**
		**pH 1**	**pH 2**	**pH 3**	**pH 1**	**pH 2**	**pH 3**		
XY	8.49 ± 0.025^c^	2.00 ± 0.014^e^	1.23 ± 0.022^e^	0.66 ± 0.003^g^	4.07 ± 0.004^d^	2.98 ± 0.002^d^	3.53 ± 0.002^a^	3.15 ± 0.004^c^	3.5 ± 0.026^d^
CP	3.36 ± 0.020^f^	2.13 ± 0.030^c^	1.74 ± 0.041^c^	2.15 ± 0.069^c^	5.53 ± 0.042^b^	3.51 ± 0.041^c^	2.92 ± 0.028^c^	3.36 ± 0.02^b^	3.77 ± 0.008^c^
WPEP	10.30 ± 0.023^a^	2.86 ± 0.023^b^	3.89 ± 0.045^a^	3.93 ± 0.045^a^	2.34 ± 0.025^f^	3.67 ± 0.025^b^	1.87 ± 0.010^e^	4.30 ± 0.025^a^	4.70 ± 0.020^b^
c-XY	8.86 ± 0.020^b^	1.49 ± 0.005^f^	1.22 ± 0.002^e^	1.04 ± 0.033^e^	4.16 ± 0.003^c^	2.38 ± 0.001^f^	3.27 ± 0.002^b^	0.88 ± 0.006^g^	5.28 ± 0.038^a^
c-CP	2.61 ± 0.017^g^	2.04 ± 0.004^d^	1.73 ± 0.031^c^	2.58 ± 0.02^b^	5.62 ± 0.006^a^	4.11 ± 0.032^a^	2.76 ± 0.006^d^	1.09 ± 0.009^f^	1.78 ± 0.013^g^
c-WPEP	7.70 ± 0.024^d^	0.54 ± 0.025^g^	1.31 ± 0.023^d^	1.15 ± 0.015^d^	1.14 ± 0.025^g^	1.30 ± 0.025^g^	1.22 ± 0.0025^f^	1.50 ± 0.004^d^	2.40 ± 0.010^f^
FOS	5.20 ± 0.015^e^	2.98 ± 0.056^a^	2.64 ± 0.073^b^	0.82 ± 0.005^f^	3.10 ± 0.008^e^	2.50 ± 0.002^e^	0.70 ± 0.001^g^	1.30 ± 0.002^e^	2.82 ± 0.012^e^

*Data are presented as mean ± standard error of the mean of three replicates. Different lowercase superscript letters in the same column denote significant differences (P < 0.05)*.

The minimal changes in hydrolysis degrees of the polysaccharide samples following digestion by saliva and gastric juice between 4 and 6 h indicated that the polysaccharide samples have a good resistance against digestion. Literature shows that pH values of gastric juice in a healthy individual ranged from 1.3 to 2.5. The pH values increase to 4.5–5.8 after eating. The ingested polysaccharides remain in the stomach for 4–6 h, and the undigested substances enter the large intestine. The undigested polysaccharides are pre-biotics in the large intestine (37). The FTIR analysis showed that the molecular structures of the polysaccharide samples had a β-D glycosidic bond. The hydrolytic actions of human digestive enzymes on carbohydrates are mainly involved in the cleavage of α-glycosidic bonds (43). Therefore, the polysaccharide samples were digestion resistant. In the simulated human digestive system, the degrees of hydrolysis of all polysaccharides were lower than 10%. The polysaccharides also have relatively stable main structures. They are hydrolysis resistant, and they are potent pre-biotics (44).

Bile salts are the functional components of bile. They are biological surfactants involved in the digestion and absorption of lipids in the small intestine. The concentrations of bile salts in the small intestine also ranged between 4 and 20 mM. The values may fall to as low as 2.6 mM in the fasted state or rise to over 15 mM in the fed state (45, 46). The effects of different bile salts on the absorption of fluid, electrolytes, and monosaccharides have been investigated in the small intestine of the experimental rats (47). The deoxycholate (1 mM) impaired absorption of water and potassium in the jejunum, but not of sodium or glucose. At higher concentrations (2.5 and 5 mM), the secretion of fluid and electrolytes occurred, and glucose and fructose absorption was impaired (48).

### Effects of Different Concentrations of Polysaccharides on Probiotic Growth

**Figure 3:**
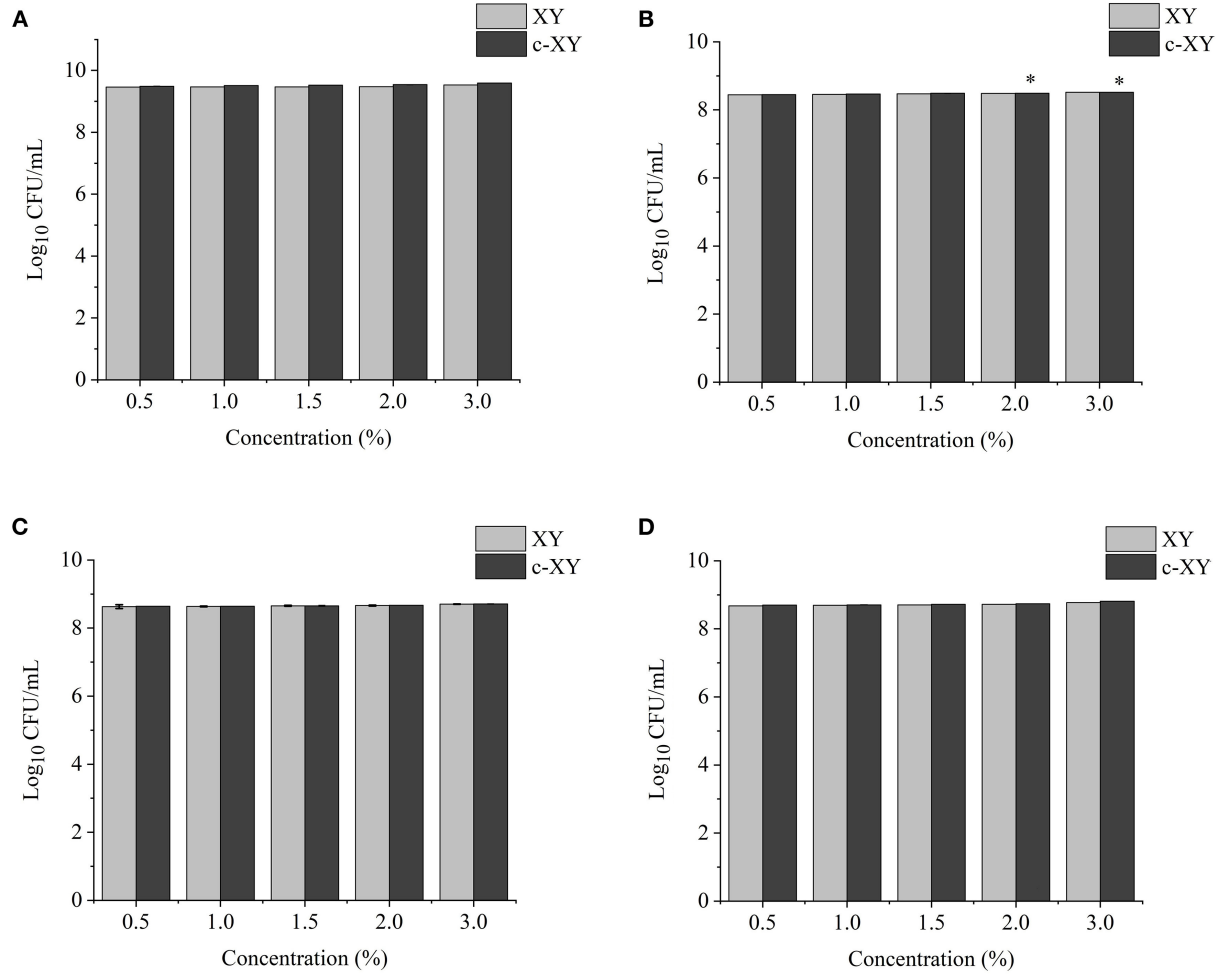
Effects of different concentrations of carboxymethylated xylan (c-XY) sample on the growth of probiotics **(A)** *L. brevis*, **(B)** *L. plantarum*, **(C)** *L. delbrueckii* subsp. *bulgaricus*, and **(D)** *S. thermophilus*. ^*^P < 0.05.

**Figure 4:**
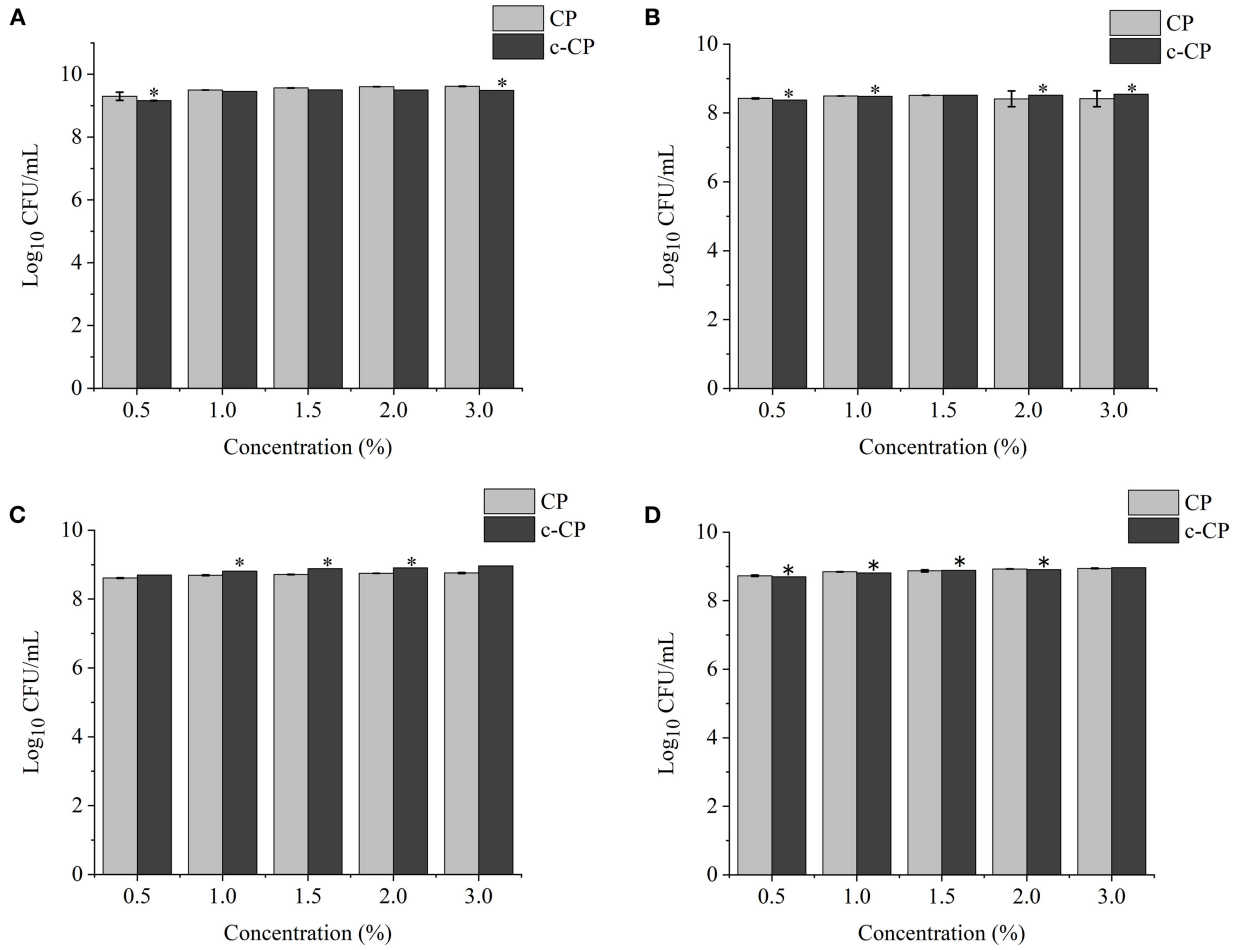
Effects of different concentrations of carboxymethylated citrus pectin (c-CP) sample on the growth of **(A)** *L. brevis*, **(B)** *L. plantarum*, **(C)** *L. delbrueckii* subsp. *bulgaricus*, and **(D)** *S. thermophilus*. ^*^P < 0.05.

**Figure 5:**
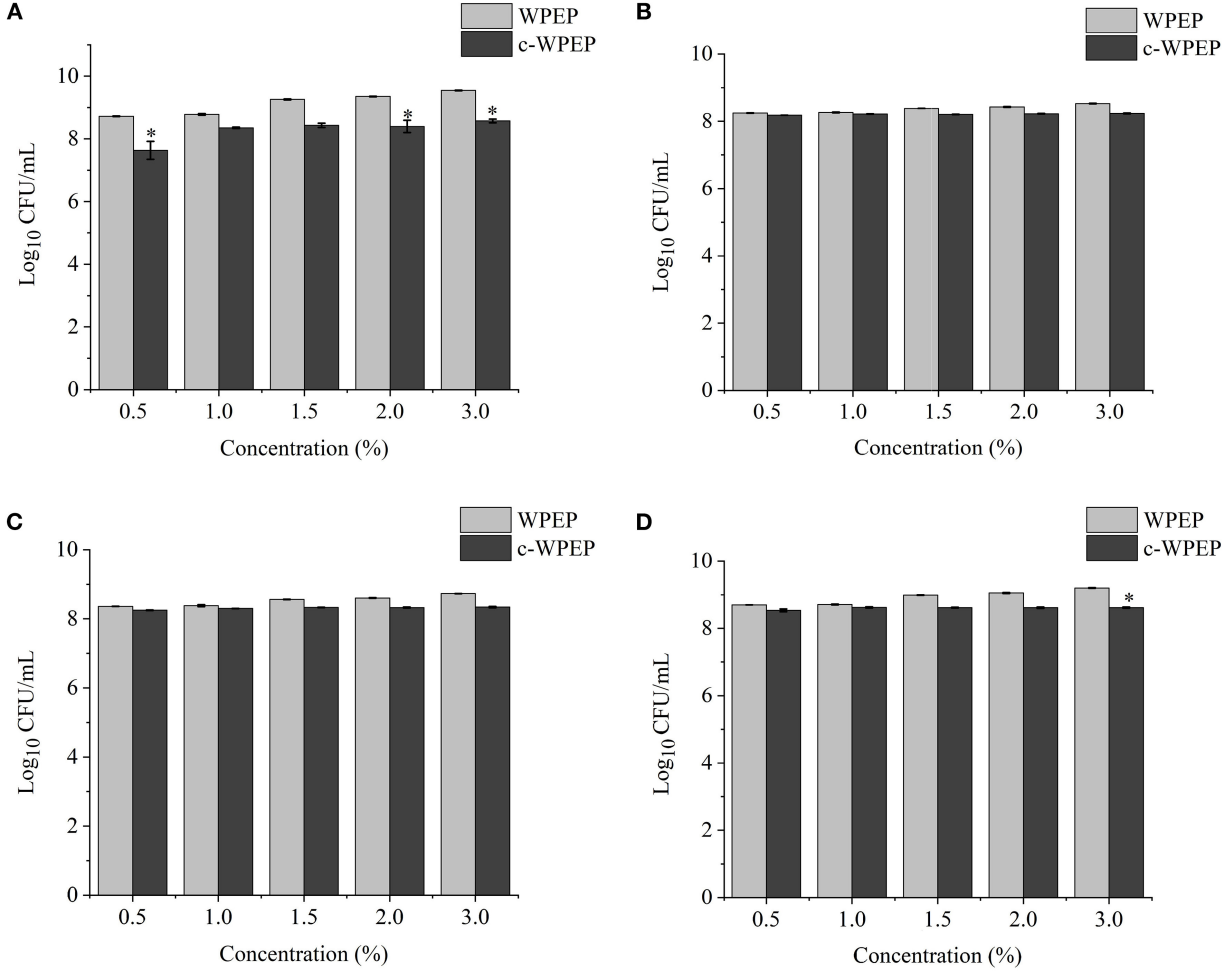
Effects of different concentrations of the carboxymethylated crude water-soluble polysaccharides of *Passiflora edulis* peel (c-WPEP) sample on the growth of **(A)** *L. brevis*, **(B)** *L. plantarum*, **(C)** *L. delbrueckii* subsp. *bulgaricus*, and **(D)** *S. thermophilus*. ^*^P < 0.05.

The effects of different concentrations of XY, CP, WPEP, c-XY, c-CP, and c-WPEP on the growth of the probiotic strains were determined (Figures 3–5). The OD values reflected the microbial counts in the fermentation broth. The changes in the values represented the growth rate of the intestinal microflora. The growth of the probiotic strains could be more accurately expressed as CFU/mL (49). The regression equations of the standard curves for *L. brevis, L. plantarum, L. delbrueckii* subsp. *bulgaricus*, and *S. thermophilus* were *y* = 4^−10^*x* + 0.007 (*R*^2^ = 0.9995), *y* = 8^−9^*x* – 1.1215 (*R*^2^ = 0.9948), *y* = 4^−9^*x* – 0.5944 (*R*^2^ = 0.9926), and *y* = 2^−9^*x* – 0.26 (*R*^2^ = 0.9958), respectively. The results showed that c-XY and c-CP promoted the growth of the probiotic strains (Figures 3, 4), especially *L. plantarum* and *L. delbrueckii* subsp. *bulgaricus*. Hence, the c-WPEP inhibited the microbial growth (Figure 5).

The finding of this study revealed that c-XY was the most effective pre-biotic in promoting the growth of *L. brevis*. It is because c-CP and c-WPEP significantly inhibited the growth of *L. brevis* and *S. thermophilus*. In this study, the polysaccharide samples promoted the growth of the *Lactobacillus* strains except for c-WPEP. The 3% c-XY had the best effect in promoting the proliferation of the probiotic strains. As shown in Figures 3, 4, c-XY and c-CP at concentrations of up to 3% effectively increased the growth of *L. plantarum* (*P* < 0.05). On the contrary, some polysaccharides were not positively correlated with the growth of the probiotic strains.

Literature demonstrated that the effect of polysaccharides isolated from the dried root of *Atractylodis macrocephalae* (42) and Fu Brick tea (37) on the growth of probiotics was not concentration-dependent. When a higher concentration of the polysaccharides was used, the growth-promoting effect weakened. It could be due to the high sugar concentration causing an increase in the osmotic pressure and accumulation of metabolites, thus limiting the proliferation of bifidobacteria.

### Effect of Optimal Concentrations of Polysaccharides on Growth Curve of Probiotics

**Figure 6:**
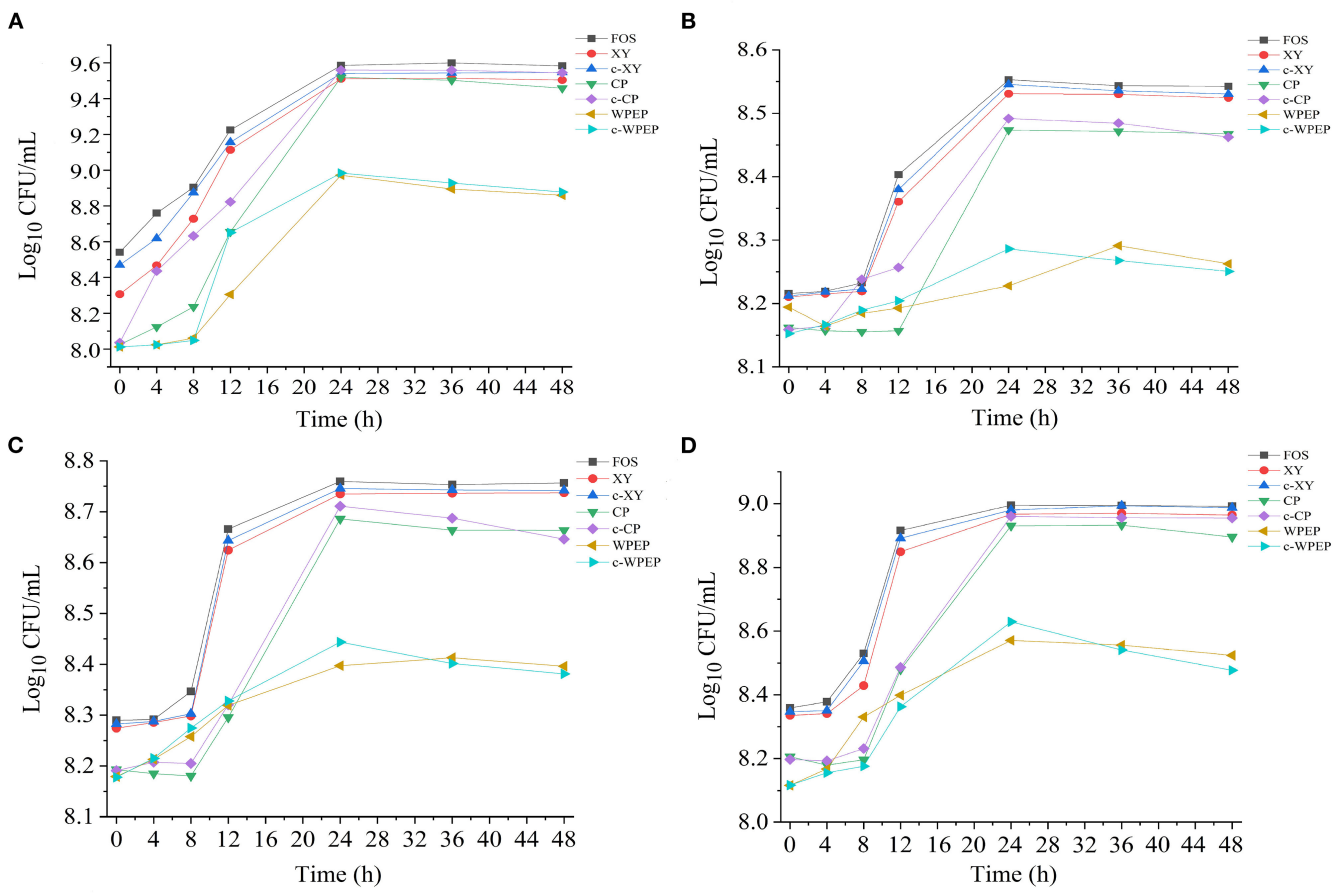
Effects of polysaccharide samples on the growth of **(A)** *L. brevis*, **(B)** *L. plantarum*, **(C)** *L. delbrueckii* subsp. *bulgaricus*, and **(D)** *S. thermophilus*.

The growth curves of the probiotic strains supplemented with the optimal concentrations of polysaccharide samples are presented in Figure 6. The addition of polysaccharide samples to the basal medium as sole carbon sources promoted microbial growth. The growth rate remained consistent for about 10 h, and the growth rate increased rapidly and reached a maximum growth rate at 24 h. After 24 h, the growth rate started to drop gradually until the end of the experiment at 48 h. The treatment with FOS showed the highest growth rates for all four probiotic strains.

The carboxymethylated polysaccharides significantly improved the growth performance of the probiotics compared with the non-carboxymethylated samples, especially for *L. brevis*. As shown in Figure 4, c-XY and c-CP had better growth-promoting effects than c-WPEP. Besides, the probiotics exposed to the carboxymethylated polysaccharides had a slightly stable growth phase after 24 h of incubation. Among the polysaccharides tested, c-XY was the most effective pre-biotic.

FOS is low molecular weight and low polymerization degree substance, and it has a better pre-biotic effect than the other polysaccharides. When FOS is added to the fermentation broth as the only energy source, it promotes the growth of the intestinal microflora. Literature also showed that FOS was the best carbon source for the proliferation of probiotics (12). Moreover, the ketone-rich FOS relieved allergic dermatitis by regulating the intestinal microflora, and it played an essential role in regulating the growth of these microorganisms (50).

The c-XY and FOS showed a similar growth performance of the four probiotics. This finding demonstrated that c-XY is a more effective pre-biotic for promoting growth of the intestinal microflora than c-CP and c-WPEP. It could be because the carboxymethylation of XY changed in its surface structure and structural bonds. Previous studies showed that the characteristics of fructose-oligosaccharides, including structural units and degree of polymerization, had a fundamental influence on its probiotic activity; c-XY and FOS might also have similar chemical information.

The growth curves of the probiotics could be divided into different phases, such as the stagnation phase, logarithmic growth phase, stationary phase, and decline phase (51). *S. thermophilus* and *L. brevis* cultivated with most polysaccharide samples had a 4 h stagnation phase, whereas *L. plantarum* and *L. delbrueckii* subsp. *bulgaricus* showed a long hour of stagnation phase before entering the logarithmic growth phase. The declining growth curves were also observed if the probiotics were kept at a prolonged period, especially after 36 h.

Studies have shown that the intestinal microflora mainly obtained nutrients from carbohydrate sources by digesting the complex polysaccharides (52). The c-WPEP showed a moderate effect in promoting the growth of the probiotics, but the result was less significant than the c-XY and c-CP. Among the three carboxymethylated polysaccharides, c-WPEP had the lowest sugar content. It indicated that c-WPEP did not provide enough energy for the growth of probiotics. Therefore, appropriate sources of pre-biotics are needed for the optimal growth of intestinal microflora (53). Also, the solubility of the c-XY and c-CP increased after carboxymethylation. The intestinal microflora could have fully utilized these carboxymethylated polysaccharides for their growth. As carboxymethylated polysaccharides have shorter chains than the non-carboxymethylated forms, the polysaccharides are easier to decompose and use by the intestinal microflora (54). Therefore, the carboxymethylated polysaccharides had a better pre-biotic effect.

## Conclusion

The carboxymethylation of XY, CP, and WPEP was successfully performed using a combination of chloroacetic acid and NaOH reactions. The successful carboxymethylation of these polysaccharides was shown by the FTIR spectra. The carboxymethylated polysaccharides had total protein content lesser than the non-carboxymethylated forms, and no significant differences were found between the polysaccharide samples. The three carboxymethylated polysaccharides resisted hydrolysis based on the assays mimicking the human digestive tract, where c-WPEP had the best resistance to digestion. The hydrolysis degrees of c-WPEP accessed by the simulated saliva, gastric juice, and small intestinal juice methods were lower than the WPEP. It showed that the carboxymethylation of WPEP increased digestion resistance in the human digestive tract. The uses of c-XY, c-CP, and c-WPEP as sole carbon sources demonstrated a variation in the effects on the growth of *L. brevis, L. plantarum, L. delbrueckii* subsp. *bulgaricus*, and *S. thermophilus*. These probiotic strains had different abilities to decompose and utilize the polysaccharides. The carboxymethylated polysaccharide samples also demonstrated better pre-biotic effects than the non-carboxymethylated samples, and c-XY had a better pre-biotic-promoting effect than c-CP and c-WPEP. The findings collectively suggested that c-XY, c-CP, and c-WPEP are potent pre-biotics that should be developed into dietary supplements for regulating *Lactobacillus* and *Streptococcus* in the human gut.

## Data Availability Statement

The original contributions presented in the study are included in the article/Supplementary Material, further inquiries can be directed to the corresponding author.

## Author Contributions

YS: conceptualization, investigation, methodology, software, and writing—original draft. HEK: supervision, formal analysis, writing—review, and editing. YG: methodology, project administration, and funding acquisition. XL: supervision, project administration, funding acquisition, writing—review, and editing. All authors contributed to the article and approved the submitted version.

## Funding

This study was supported by the National Natural Science Foundation of China (31860251), the Guangxi Science & Technology Program (AD20297088), and the Guangxi Key Laboratory of Electrochemical and Magnetochemical Functional Materials (EMFM20211104 and EMFM20211102).

## Conflict of Interest

The authors declare that the research was conducted in the absence of any commercial or financial relationships that could be construed as a potential conflict of interest.

## Publisher's Note

All claims expressed in this article are solely those of the authors and do not necessarily represent those of their affiliated organizations, or those of the publisher, the editors and the reviewers. Any product that may be evaluated in this article, or claim that may be made by its manufacturer, is not guaranteed or endorsed by the publisher.
