# Supplementary material for: In vitro Assessment of Chemical and Pre-biotic Properties of Carboxymethylated Polysaccharides From Passiflora edulis Peel, Xylan, and Citrus Pectin
Source: Front Nutr. 2021 Dec 3;8:778563. doi: 10.3389/fnut.2021.778563 (PMC8678565; doi:10.3389/fnut.2021.778563)
Supplement: Supplementary file 1 [file Data_Sheet_1.docx]

**Supplementary Figures**

**
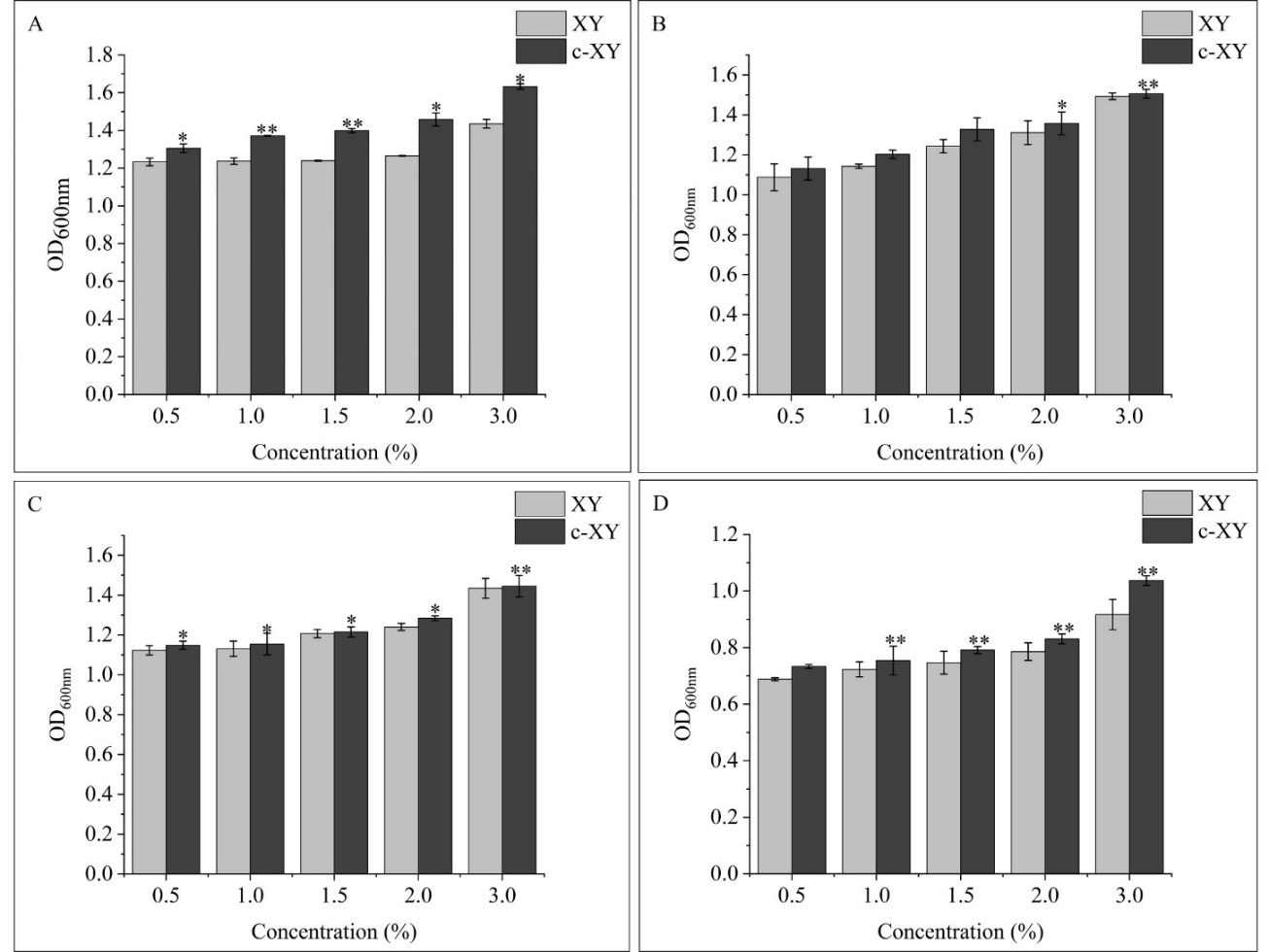
**

**Figure S1. OD values of different concentrations of carboxymethylated xylan (c-XY) sample on the growth of probiotics (A) L. brevis, (B) L. plantarum, (C) L. delbrueckii subsp. bulgaricus, and (D) S. thermophilus**

**
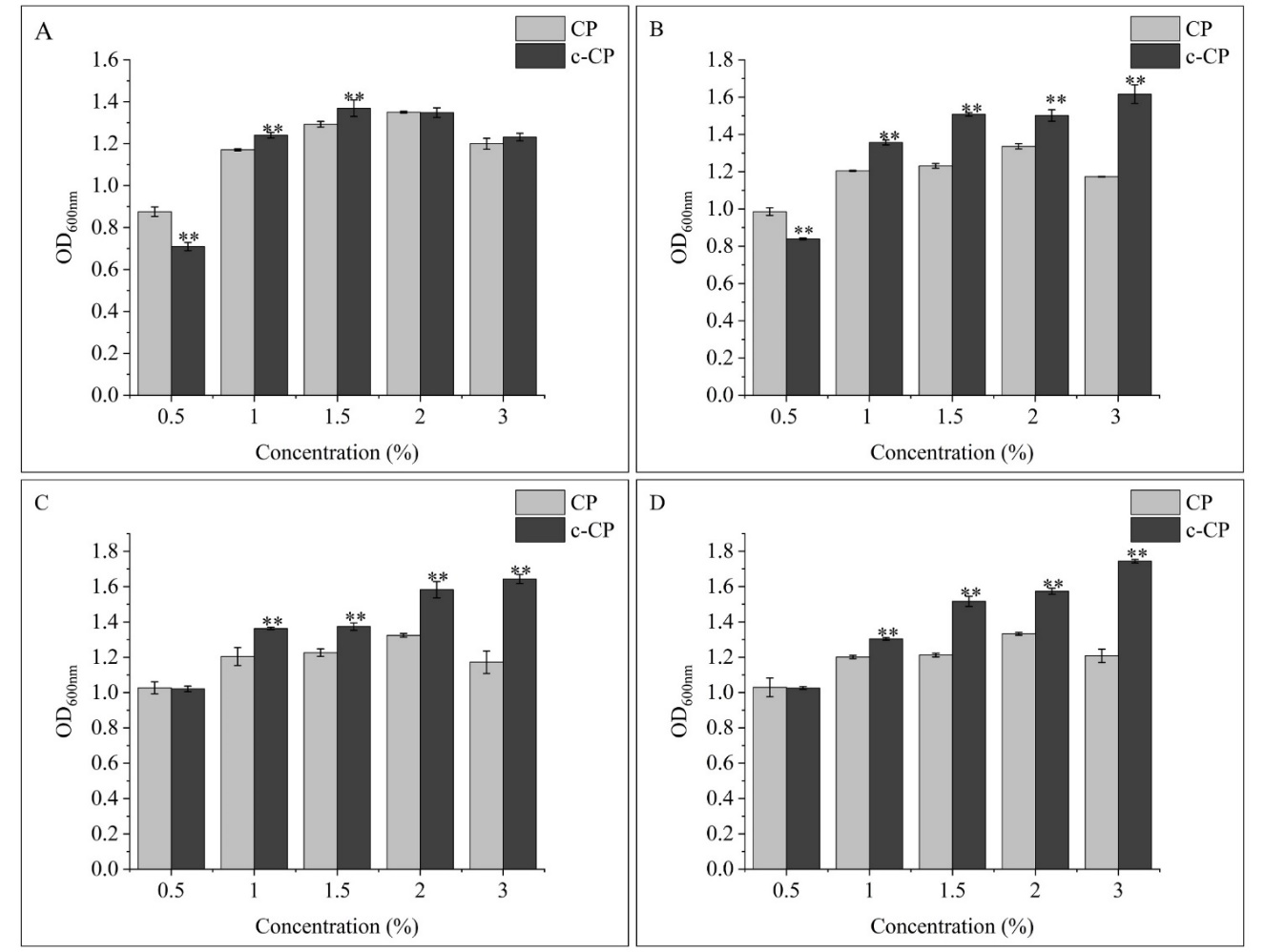
**

**Figure S2. OD values of different concentrations of carboxymethylated xylan (c-CP) sample on the growth of probiotics (A) L. brevis, (B) L. plantarum, (C) L. delbrueckii subsp. bulgaricus, and (D) S. thermophilus**

**
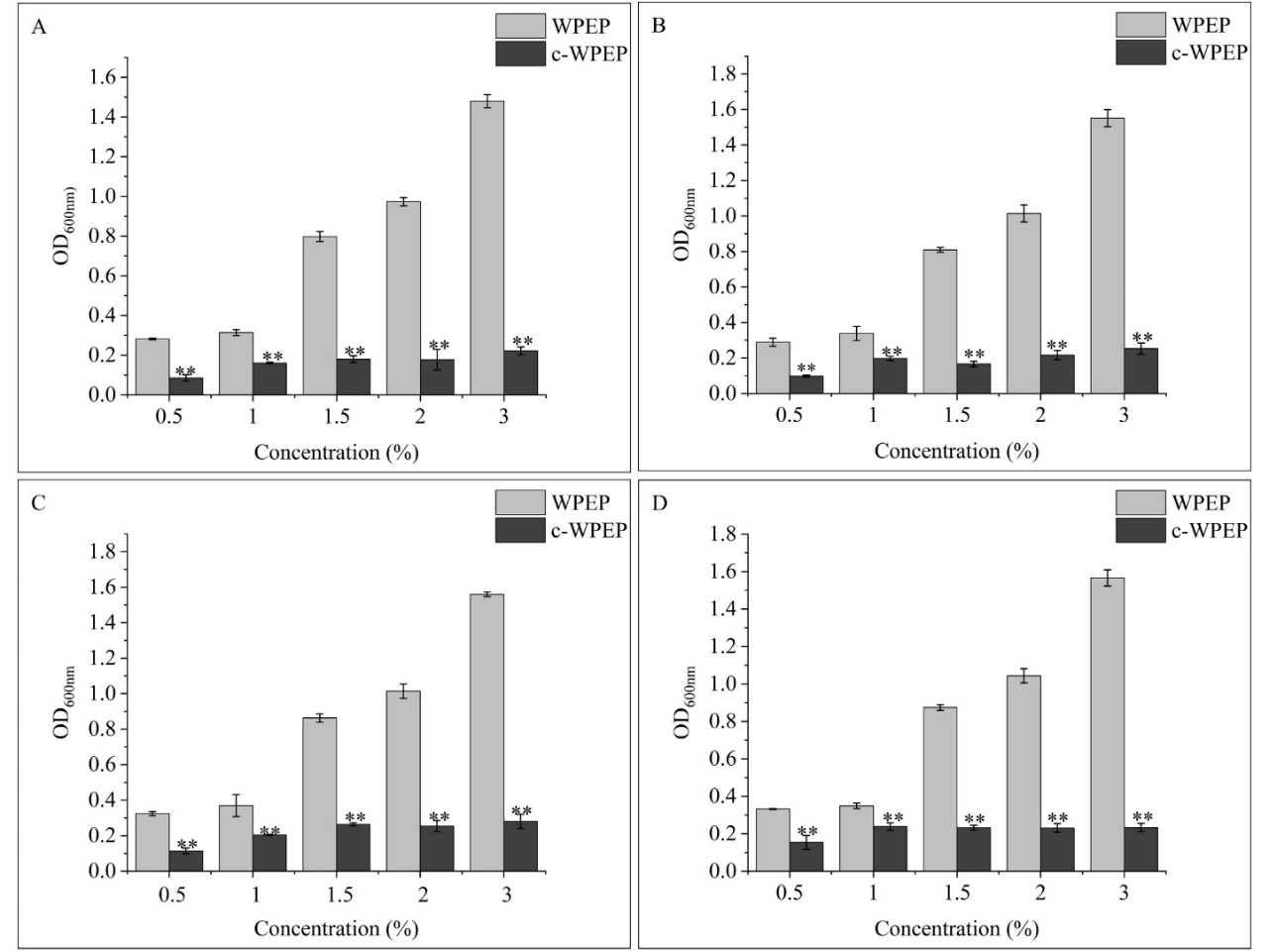
**

**Figure S3. OD values of different concentrations of carboxymethylated xylan (c-WPEP) sample on the growth of probiotics (A) L. brevis, (B) L. plantarum, (C) L. delbrueckii subsp. bulgaricus, and (D) S. thermophilus**

**
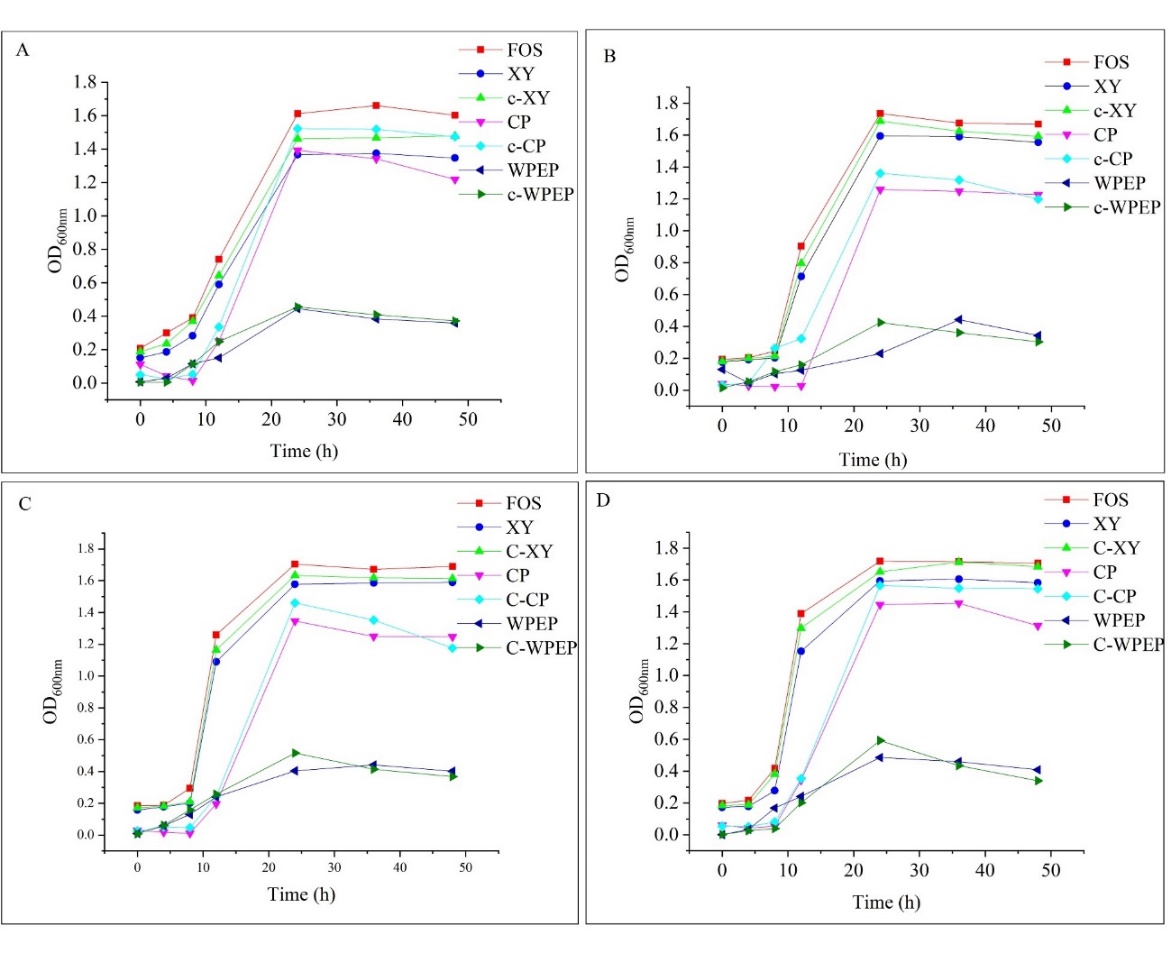
**

**Figure S4. polysaccharide samples on the growth of (A) L. brevis, (B) L. plantarum, (C) L. delbrueckii subsp. bulgaricus, and (D) S. thermophilus.**
